# Morphology, DNA barcoding and seasonal occurrence of *Ergasilus lizae* Krøyer, 1863 (Copepoda: Ergasilidae) parasitizing mullets from northwestern Mexico

**DOI:** 10.1007/s11230-024-10179-8

**Published:** 2024-08-09

**Authors:** Francisco Neptalí Morales-Serna, Selena Camacho-Zepeda

**Affiliations:** 1 Instituto de Ciencias del Mar y Limnología, Universidad Nacional Autónoma de México, Mazatlán, Mexico; 2 Posgrado en Ciencias en Recursos Acuáticos, Facultad de Ciencias del Mar, Universidad Autónoma de Sinaloa, Mazatlán, Mexico

## Abstract

*Ergasilus lizae* Krøyer, 1863 is a parasitic copepod known to infect mullets (Mugilidae) in different parts of the world. It was originally reported from the east coast of North America, but the original description lacks enough detail, making identification with this information difficult. In this study, we provide a redescription of *E. lizae* found on *Mugil curema* Valenciennes and *M. cephalus* Linnaeus, caught in two coastal lagoons of northwestern Mexico during two climatic seasons: warm/rainy and cold/dry. The prevalence of this parasite was higher in the warm season than in the cold season. To facilitate the species identification, new sequences of the barcoding gene (COI mtDNA) of *E. lizae* were generated and compared against unpublished sequences of *E. lizae* available in the Barcode of Life Database (BOLD). Our results suggest that the sequences of BOLD possibly belong to a species misidentified as *E. lizae*.

## Introduction

The Ergasilidae Burmeister, 1835 (Crustacea: Copepoda: Cyclopoida) is a family of copepods whose adult females are commonly found parasitizing teleosts, while adult males and larval stages are planktonic in all salinity regimes (Amado et al., 1995). Within this family, *Ergasilus* von Nordmann, 1832 is the most speciose genus with 163 species (Walter & Boxshall, 2024). *Ergasilus lizae* Krøyer, 1863, considered a cosmopolitan parasite of mullets, was originally reported from *Mugil curema* Valenciennes from New Orleans (east coast of USA), and then from other parts of the world, including Italy, Egypt, France, Israel, Turkey, Chile, Brazil, Australia and Mexico (Causey, 1960; Roberts, 1970; Kabata, 1992; Knoff et al., 1994; El-Rashidy, 1999; Özer & Kirca, 2013). As the original morphological description is inadequate, Kabata (1992) redescribed *E. lizae* based on specimens collected from *Mugil cephalus* Linnaeus and *Trachystoma petardi* (Castelnau) caught in Brisbane River, Australia. However, based on lectotype material deposited in Copenhagen Zoological Museum, El-Rashidy (1999) argued that the Australian material revised by Kabata (1992) is not conspecific with *E. lizae*. In his doctoral thesis, El-Rashidy (1999) redescribed *E. lizae* with detailed illustrations. Unfortunately, El-Rashidy’s (1999) work was never published.

In northwestern Mexico, *E. lizae* was reported by Causey (1960) from *M. cephalus* collected in Mazatlán, Sinaloa state and San Blas, Nayarit state, but comments related to the species identification were not provided. As far as we know, the presence of *E. lizae* in Mexican fishes has not been confirmed in previous studies other than Causey (1960). In fact, ergasilids remain largely unstudied in Central America and Mexico and many reports are referred to as *Ergasilus* sp. (Suárez-Morales & Santana-Piñeros, 2008; Jiménez-García & Suárez-Morales, 2017).

The identification of ergasilids could be more precise with the integration of morphological and molecular data, particularly the barcoding gene, cytochrome c oxidase subunit I (COI mtDNA). However, the number of COI sequences is currently limited to very few species of *Ergasilus*, in part due to difficulties in the amplification process (Míč et al., 2023). Recently, a comparative analysis of COI sequences of ergasilids could only include sequences for three known species of *Ergasilus* due to the unavailability of verifiable published sequences (Fikiye et al., 2023). One of those three species was *E. lizae* whose unpublished sequences are available in the Barcode of Life Database (BOLD). These sequences were generated from specimens found in *Fundulus diaphanus* (Lesueur) collected in Richelieu River, Quebec, Canada.

In the present study, we provide morphological data of specimens of *E. lizae* collected from *M*. *curema* and *M. cephalus* from two coastal lagoons in Sinaloa state, northwestern Mexico. Newly generated COI sequences of *E. lizae* were compared with sequences retrieved from BOLD. Additionally, the prevalence and intensity of the infection in warm and cold seasons were assessed.

## Materials and methods

### Fish and parasite collection

**Fig. 1:**
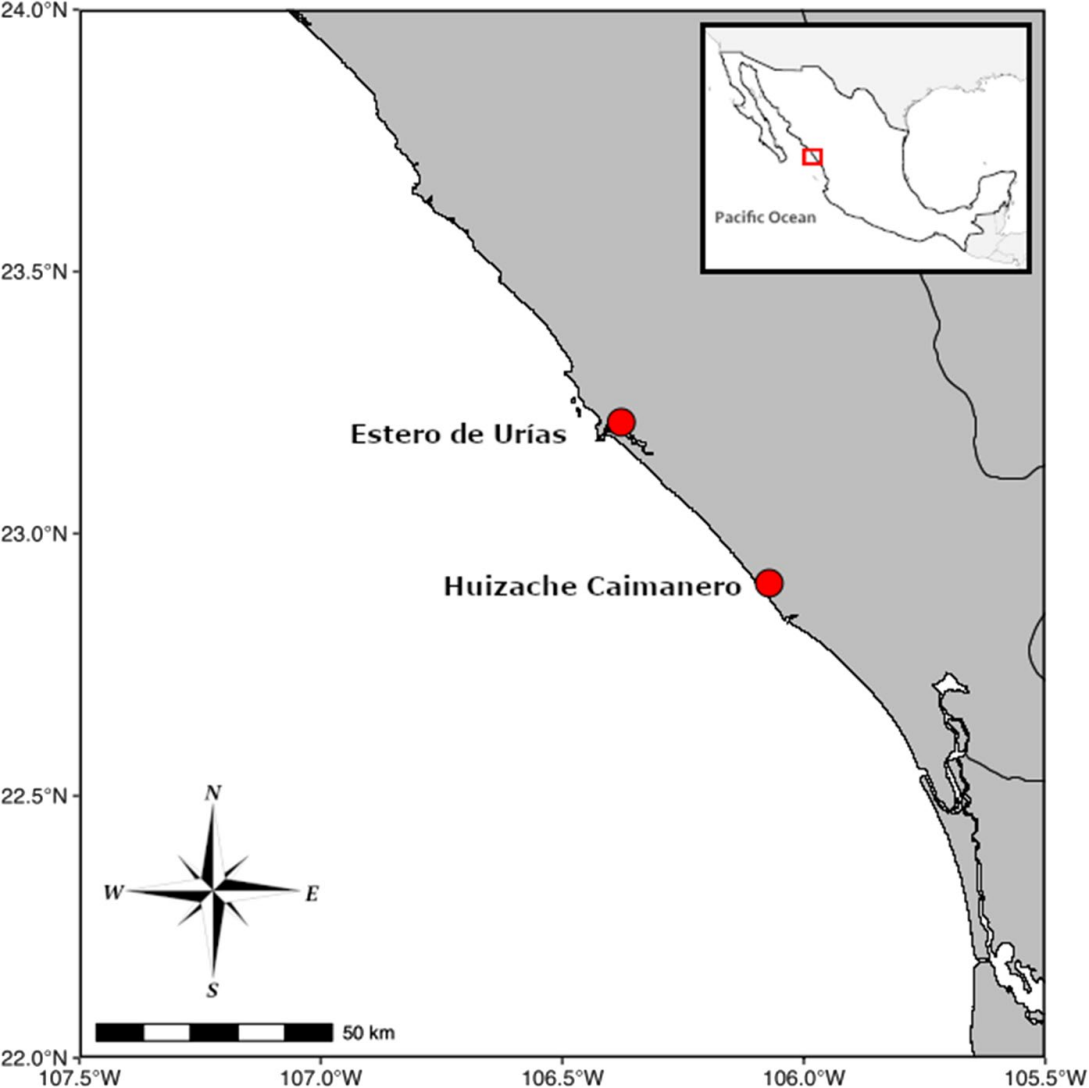
Map indicating the location of Urías estuary and Huizache-Caimanero lagoon, in northwestern Mexico, where individuals of *Mugil curema* and *M. cephalus* were obtained for parasitological analysis

Ninety-three specimens of *M. curema* were purchased from local fishermen in two seasons. These fish were caught during August 2022 (warm/rainy season, with an average temperature of 32 °C) and January 2023 (cold/dry season with an average temperature of 19 °C) in Urías estuary and Huizache-Caimanero coastal lagoon, southern Sinaloa state, northwestern Mexico (Figure 1, Table 1). Additionally, 21 specimens of *M. cephalus* were purchased from fisherman, caught during August 2023 in Huizache-Caimanero. Both lagoons have multiple anthropogenic stressors (Martínez-Salcido et al., 2018). Urías estuary has been strongly impacted by sewage and industrial effluents from the Mazatlán harbor, whereas Huizache-Caimanero lagoon has mostly been impacted by agriculture. The geographical distance between these lagoons is approximately 45 km.

**Table 1:** Samples of *Mugil curema* and *M. cephalus* obtained from Urias Estuary (UE) and Huizache-Caimanero lagoon (HC) during two seasons.

Fish host	Locality	Season	n	TL (cm)
*Mugil curema*	UE	Warm/rainy	30	30.2 ± 3.4
		Cold/dry	26	28.6 ± 2.1
	HC	Warm/rainy	20	31.2 ± 1.8
		Cold/dry	17	29.1 ± 1.3
*Mugil cephalus*	HC	Warm/rainy	21	34.7 ± 1.5

TL = Mean ± SD of the fish total length.

In the laboratory, fish were identified to species level following Froese and Pauly (2023). The total length (cm) of each fish was recorded. Gills were removed and examined for the presence of parasitic copepods with the aid of a dissecting microscope (Motic, Richmond, BC, Canada). Copepods were counted, removed using fine needles and preserved in 96% ethanol.

### Morphological analysis

Copepods were cleared in 85% lactic acid for a few minutes and then examined under a Leica DMLB microscope. The total body length, from the anterior margin of the prosome to the posterior margin of caudal rami (excluding caudal setae), was measured using an ocular micrometer. Drawings of the entire body and dissected appendages, temporarily mounted in slides with lactic acid, were made with the aid of a drawing tube. Final illustrations were digitally inked using INKSCAPE 1.0. The voucher specimens were deposited in the Copepoda collection of the Instituto de Ciencias del Mar y Limnología, Unidad Académica Mazatlán (ICML-EMUCOP), Sinaloa, Mexico.

### Molecular analysis

Total DNA was extracted using the Jena Bioscience kit, following the manufacturer’s instructions (Jena Bioscience, Jena, Germany). Amplification and sequencing of the COI gene were carried out using invertebrate universal “Folmer” primers LCO1490 (5′-GGT CAA CAA ATC ATA AAG ATA TTG G-3′) and HCO2198 (5′-TAA ACT TCA GGG TGA CCA AAA AAT CA-3′). Thermal cycling conditions for amplification reactions were 94 °C for 2 min, 30 cycles at 94 °C for 1 min, 48 °C for 40 s, 72 °C for 2 min and a final extension at 72 °C for 7 min. PCR products were sequenced using an ABI 3730xl Genetic Analyzer (Thermo Fisher Scientific, Waltham, Massachusetts, USA) at the Laboratorio Nacional de la Biodiversidad, Instituto de Biología, Universidad Nacional Autónoma de México.

Forward and reverse sequences were assembled using Geneious 5.1.5 (Biomatters Ltd. Auckland, New Zealand). The newly generated sequences were compared against sequences of *E. lizae* retrieved from BOLD and the sequences of other 10 species of *Ergasilus* available in GenBank (Table 2). Sequences were aligned using MUSCLE 3.8 (Edgar, 2004) with default parameters, and trimmed using trimAL 1.2 (Capella-Gutiérrez et al., 2009) with the parameter gappyout. The best evolutive model, GTRGAMMA, was selected with the program ModelFinder (Kalyaanamoorthy et al., 2017). A phylogenetic tree was constructed under Maximum likelihood (ML) with RAxML 8.2.12 (Stamatakis, 2014). Nodal support for the tree was assessed through bootstrap analysis with 1000 replicates. *Lernaea cyprinacea* (Linnaeus, 1758) (Copepoda: Cyclopoida: Lernaeidae) was used as the outgroup. Pairwise sequence similarity was estimated with Clustal-Omega 2.1 (Sievers et al., 2011). GenBank accessions of the COI sequences generated for *E. lizae* of the present study are given in Table 2.

**Table 2:** Species of *Ergasilus* included in the comparative analysis of COI sequences.

Species	Host	Locality	Accession	Reference
*Ergasilus caeruleus*	Free-living	Great lakes	OP830265	Unpublished
*Ergasilus centrarchidarum*	Free-living	Great lakes	OP830192	Unpublished
*Ergasilus chautauquaensis*	Free-living	Great lakes	OP830262	Unpublished
*Ergasilus jaraquensis*	–	Brazil	MF651988	Unpublished
*Ergasilus lizae*	*Fundulus diaphanus*	Canada	ECTCR024-14, ECTCR025-14	Unpublished
***Ergasilus lizae***	***Mugil curema***	**Northwestern Mexico**	**PP239046, PP239047, PP239048**	**Present study**
*Ergasilus luciopercarum*	Free-living	Great lakes	OP830347	Unpublished
*Ergasilus megaceros*	Free-living	Great lakes	OP830321	Unpublished
*Ergasilus mirabilis*	*Clarias gariepinus*	South Africa	OR448769	Fikiye et al. (2023)
*Ergasilus versicolor*	Free-living	Great lakes	OP830335	Unpublished
*Ergasilus wilsoni*	Free-living	South Korea	KR049036	Baek et al. (2016)
*Ergasilus* sp.	–	Caribbean, Mexico	MZ517943	Unpublished
*Lernaea cyprinacea*	*Carassius auratus*, *Cyprinus carpio*, *Chanodichthys ilishaeformis*	China	MH982220	Hua et al. (2019)

All the accession numbers correspond to GenBank records, except for those of *E. lizae* from Canada, which correspond to BOLD records (https://www.boldsystems.org/ accessed on November 30, 2023).

### Occurrence

The prevalence and median intensity of infection (sensu Bush et al., 1997; Reiczigel et al., 2019), with confidence intervals (CI) at 95%, were calculated and compared between seasons with Fisher’s exact test and bootstrap t-test (with 1000 replications), respectively, using the software Quantitative Parasitology (Reiczigel et al., 2019).

## Results

### Morphology

Order Cyclopoida Burmeister, 1834

Family Ergasilidae Burmeister, 1835

Genus *Ergasilus* von Nordmann, 1832

*Ergasilus lizae* Krøyer, 1863

*Host*: *Mugil curema* Valenciennes and *M. cephalus* Linnaeus (Mugilidae).

*Locality*: Urías estuary (23°12’32.6” N, 106º23’35.4” W), and Huizache-Caimanero lagoon (22°54’19.6” N, 106°03’42” W), Sinaloa state, northwestern Mexico.

*Material examined*: 20 ovigerous females from *M. curema* caught in Urias estuary, and eight ovigerous females from *M. cephalus* caught in Huizache-Caimanero lagoon, of which, 10 were dissected, 3 were used for DNA extraction, and 15 were designed as voucher specimens (ICML-EMUCOP 160822-01, 100123-01, and 120123-01).

#### Redescription of adult female

**Fig. 2:**
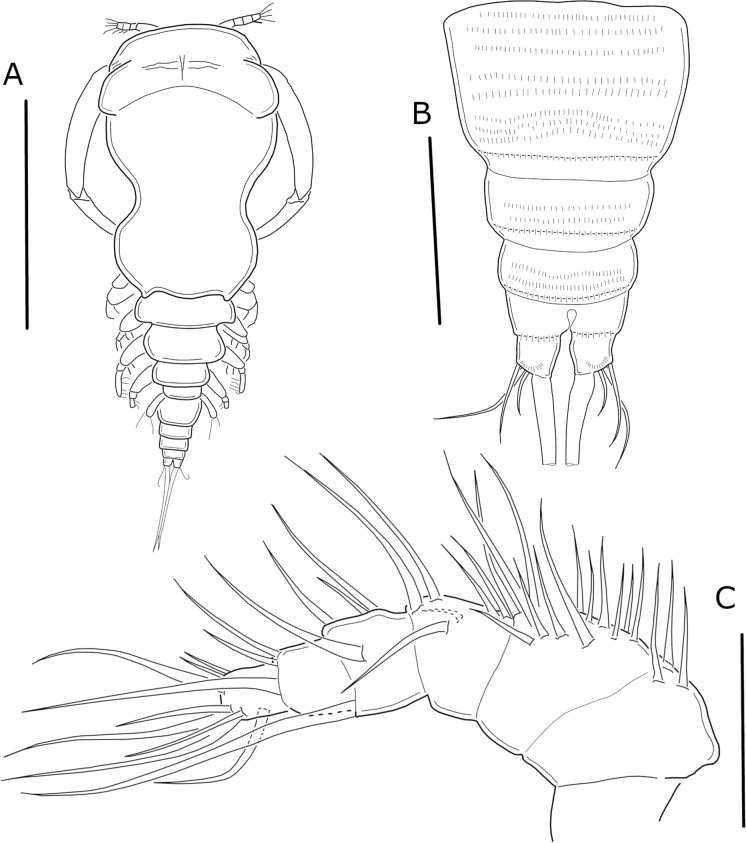
*Ergasilus lizae* Krøyer, 1863. Adult female. **A** Habitus, dorsal view; **B** Urosome, ventral view; **C** Antennule. Scale bars: **A** = 500 µm; **B** = 100 µm; **C** = 50 µm

Body cyclopiform (Figure 2A), length ranging from 0.7 to 1.1 mm (mean of 0.82 mm, n = 10) from anterior margin of prosome to posterior margin of caudal rami. Prosome 5-segmented, comprising semi-oval cephalosome and 4 pedigerous somites gradually tapering posteriorly; with dorsal depression between cephalosome and first pedigerous somite, forming bilobular-shaped prosome. Cephalosome wider than first pedigerous somite. Urosome narrow, comprising short fifth pedigerous somite, genital double-somite, and three free somites. Urosomites with rows of spinules on ventral surface (Figure 2B). Caudal rami subquadrate, each ramus bearing 4 setae—the innermost seta noticeably thicker and longer than the other setae (Figure 2B).

Antennule (Figure 2C) 6-segmented, bearing simple setae. Second to fifth segments gradually tapering distally. Setal formula from proximal to distal segments: 3, 13, 6, 5, 3, 8.

**Fig. 3:**
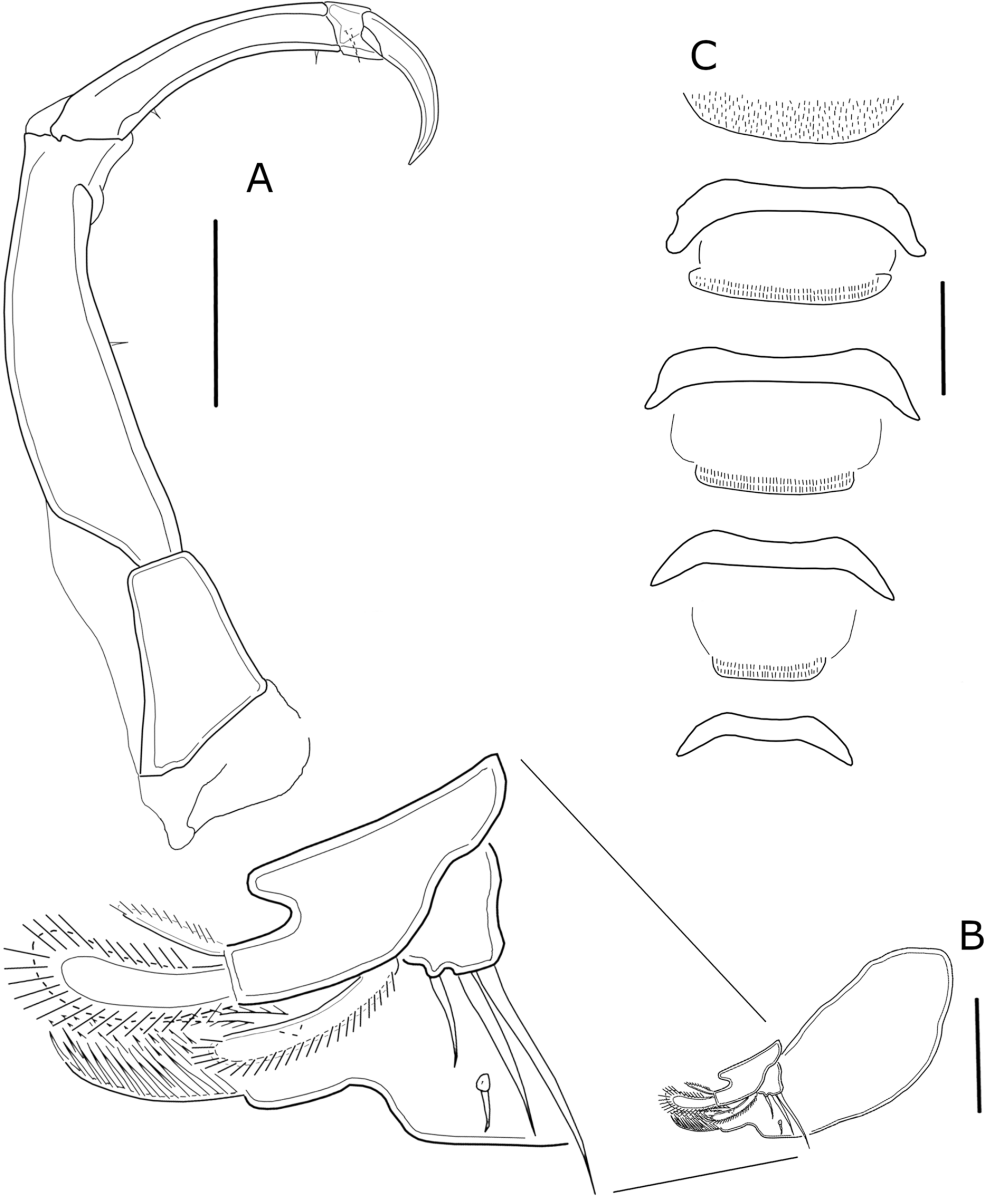
*Ergasilus lizae* Krøyer, 1863. Adult female. **A** Antenna; **B** Mouth parts including mandible, maxillule and maxilla, ventral view; **C** Interpodal plates of legs 1 to 4, ventral view. Scale bars: **A** = 100 µm; **B** and **C** = 50 µm

Antenna (Figure 3A) 4-segmented, comprising short coxobasis, 3-segmented endopod, and terminal claw. Second segment (first endopodal segment) longest, twice as long as the coxobasis, bearing 1 minute seta midway inner margin. Third segment (second endopodal segment) elongated, about 1/3 the length of preceding segment, with 1 proximal and 1 subdistal minute seta on concave inner margin. Fourth segment (third endopodal segment) smallest, with 1 small seta. Terminal claw curved, about 1/2 the length of the second endopodal segment.

Mouth parts (Figure 3B) comprising mandible, maxillule and maxilla. Mandible consisting of 3 blades of which anterior blade smallest and with teeth on anterior margin, middle blade wide with teeth anteriorly and posteriorly, posterior blade elongated with teeth on posterior margin. Maxillule lobate, with 3 unequal setae. Maxilla 2-segmented; distal segment bearing sharp teeth anteriorly and 1 spinulate seta.

**Fig. 4:**
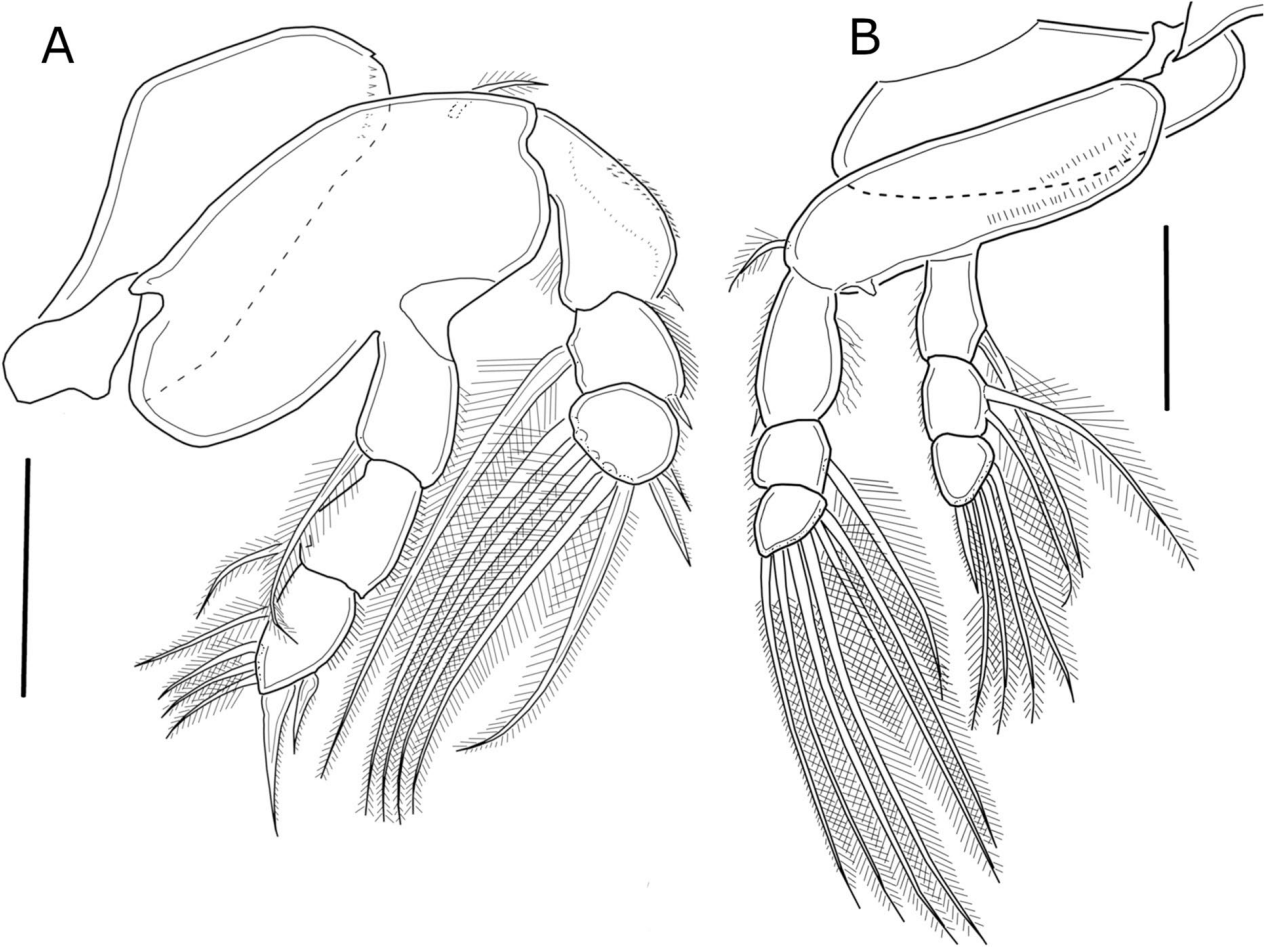
*Ergasilus lizae* Krøyer, 1863. Adult female. **A** Leg 1, anterior view; **B** Leg 2, anterior view. Scale bars: **A** and **B** = 50 µm

**Fig. 5:**
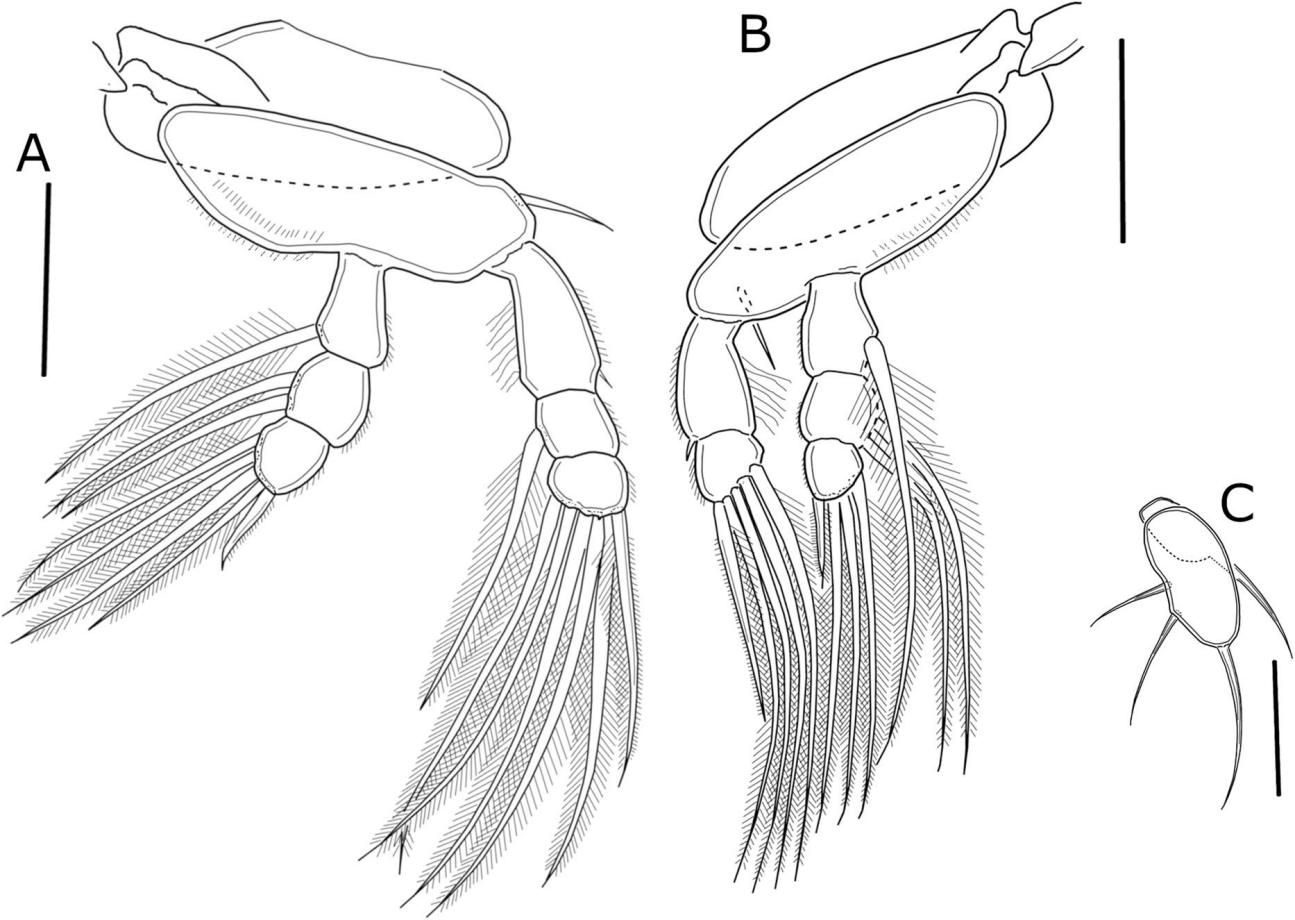
*Ergasilus lizae* Krøyer, 1863. Adult female. **A**, Leg 3, anterior view; **B**, Leg 4, anterior view; **C**, Leg 5. Scale bars: **A**–**C** = 50 µm

Swimming legs 1 to 4 (Figs. 4A, B, 5A, B) biramous. All rami 3-segmented, except for 2-segmented exopod of leg 4 (Figure 5B). All legs with row of spinules on outer margin of both rami and setules on inner margin of first exopodal segments. Second leg basis with spiniform process between insertion of endopodal and exopodal rami. Interpodal plates with rows of spinules on posterior margin (Figure 3C). Armature of legs as follows (Roman and Arabic numerals for spines and setae, respectively).

**Table:** 

	Coxa	Basis	Exopod	Endopod
Leg 1	0-0	1-0	I-0; I-1; III,4	0-1; 0-1; II,4
Leg 2	0-0	1-0	I-0; 0-1; I,5	0-1; 0-2; I,4
Leg 3	0-0	1-0	I-0, 0-1; I,5	0-1; 0-2; I,4
Leg 4	0-0	1-0	I-0; I,4	0-1; 0-2; I,3

Leg 5 (Figure 5C) 2-segmented, consisting of short protopodal segment with 1 outer seta, and free exopodal segment with 1 distal and 2 subdistal setae.

### DNA barcoding

**Fig. 6:**
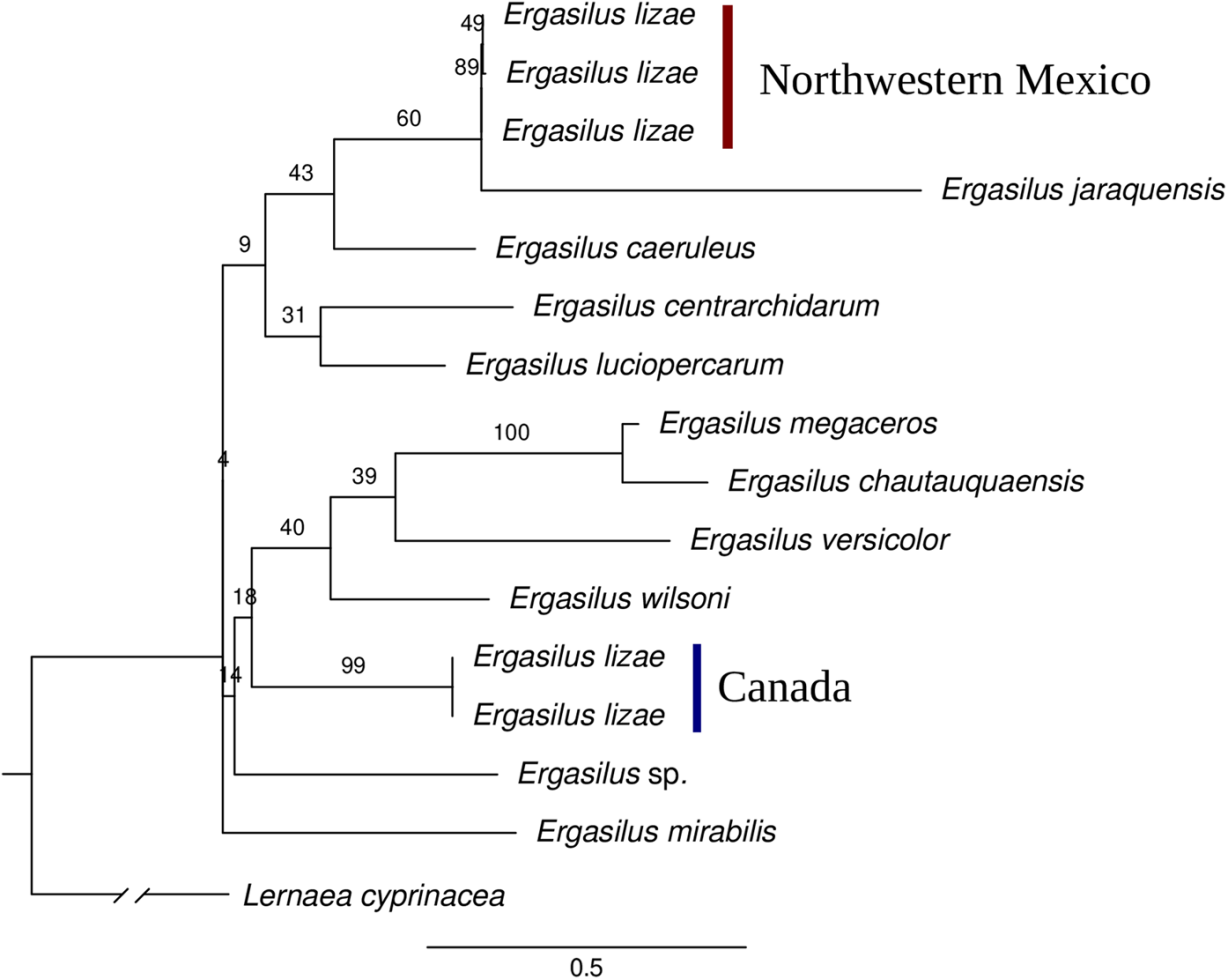
Maximum likelihood tree based on COI sequences of copepods of the genus *Ergasilus*. The branch labeled as Northwestern Mexico contains the newly generated sequences for *E. lizae*. Bootstrap support is displayed at the nodes. The cyclopoid copepod *Lernaea cyprinacea* (Linnaeus, 1758) was used as the outgroup

Three COI sequences were generated for *E. lizae* from northwestern Mexico. These sequences were aligned with two sequences of *E. lizae* from Canada, 10 sequences of other ergasilids and one sequence of *L. cyprinacea* as the outgroup, resulting in an alignment of 651 bases. In the ML tree (Figure 6), *E. lizae* from northwestern Mexico was grouped with *E. jaraquensis* from Brazil, whereas *E. lizae* from Canada appeared in a separate branch; however, the bootstrap values were very low. The specimens of *E. lizae* from northwestern Mexico showed an intraspecific sequence similarity 99%, and showed a similarity of 80% with both *E. lizae* from Canada and *E. jaraquensis*.

### Occurrence

The prevalence of *E. lizae* on *M. curema* was significantly higher in the warm than in the cold season in both coastal lagoons. In Urías estuary, the prevalence was 62% (CI 43–78%) and 12.5% (CI 3.5–30%) in the warm and the cold season, respectively, whereas in Huizache-Caimanero lagoon it was 55% (CI 32–76%) and 12% (CI 2–34%) in the warm and the cold season, respectively, respectively (P < 0.05). In both lagoons, the median intensity was 2 and 4 (CI 1–6) copepods per fish in the warm and cold season, respectively. This difference was not significant (P > 0.05). This analysis was not performed for *M. cephalus* because samples were not obtained during the cold season.

## Discussion

### Morphology

The morphological characteristics of the specimens examined in the present study agree with those of *E. lizae* as redescribed by El-Rashidy (1999), who examined the lectotype material (CRU-7091) deposited in Copenhagen Zoological Museum. We agree with El-Rashidy (1999) that the material redescribed by Kabata (1992) is not conspecific with *E. lizae*. Kabata’s material lacks the depression between the cephalosome and first pedigerous somite, the spinulate seta on the basis of the maxilla, the surface ornamentation on the interpodal plates, and the outer spine on the second exopodal segment of leg 1, all of which are typical for *E. lizae*. Additionally, the maxillule has 3 outer setae and 1 medial process in *E. lizae* but only 2 outer setae are present in Kabata’s material. Also, the second segment of leg 5 has 3 setae in *E. lizae* but only 2 in Kabata’s material. Regarding the habitat, our specimens occurred on fish caught in marine waters, whereas the Australian specimens were taken in freshwater habitats. According to Jiménez-García and Suárez-Morales (2017), *E. lizae* seems to be restricted to marine habitats.

*Ergasilus lizae* morphologically resembles *E. arthrosis* Roberts, 1969, *E. atafonensis* Amado & Rocha, 1996, and *E. parabahiensis* El-Rashidy & Boxshall, 1999 (El-Rashidy, 1999). Compared with *E. arthrosis*, *E. lizae* has a shorter antennary claw and a distal segment of the antenna with 8 setae instead of 5. Additionally, *E. arthrosis* appears to be restricted to freshwater habitats (Jiménez-García & Suárez-Morales, 2017). *Ergasilus atafonensis* lacks of spinulate seta of the maxilla and spiniform process on the basis of the second leg. In *E. parabahiensis*, the antennal segments are shorter and the spiniform process on the basis of the second leg is absent. According to El-Rashidy (1999), *E. lizae* also resembles *E. tissensis* D'yachenko, 1969; however, the mouth parts of this species were not described.

### DNA barcoding

The separation between the newly generated sequences and the sequences of *E. lizae* from Canada indicated by the ML tree suggests that these belong to different species. As stated above, *E. lizae* seems to be restricted to marine habitats. However, the Canadian specimens were isolated from a freshwater fish, *F. diaphanus*. The Canadian specimens were sequenced by Dr. Sean Locke (currently at the University of Puerto Rico). According to Dr. Locke (pers. comm.), the specimens that he sequenced possibly were misidentified as *E. lizae* since there was no rigorous morphological examination. The limited number of published of COI sequences for *Ergasilus* hinders the ability to perform a comprehensive phylogenetic analysis. Despite these limitations, the sequences provided herein represent a valuable contribution to the genetic database of *Ergasilus* species and lay the groundwork for future research. Expanding the dataset and incorporating additional genetic information will enhance our understanding of the evolutionary relationships and diversity of this important group of parasitic copepods.

### Occurrence

The higher prevalence of *E. lizae* observed during the warm season agrees with the seasonal occurrence reported for other ergasilids. For instance, studies of *Ergasilus sieboldi* von Nordmann, 1832 on pikeperch, and *Sinergasilus major* (Markevich, 1940) and *S. polycolpus* (Markevich, 1940) on farmed carp reported higher infection levels during summer (Molnár & Székely, 1997; Nie & Yao, 2000). This was probably due to the slower formation of eggs with the decrease of water temperature (Paperna & Zwerner, 1976). As typically observed in marine ectotherms, within an optimal range, water temperature has a negative relationship with development times, body size, and reproductive outputs of parasitic copepods (Samsing et al., 2016). Thus, generation times are shorter at high temperatures, which can be reflected in higher infection levels during warm seasons; nonetheless, females are bigger and produce more eggs at low temperatures, a situation that also can result in high infection levels during cold seasons. For instance, Hogue et al. (1993) observed that *E. nerkae* Roberts, 1963 was more prevalent on longnose sucker in winter.

## Conclusions

Based on a detailed morphological analysis, this study confirms the presence of the parasitic copepod *E. lizae* on mullets (*M. cephalus* and *M. curema*) from northwestern Mexico. Additionally, COI sequences for *E. lizae* are provided for the first time. Given that *E. lizae* is a cosmopolitan species, our findings could enhance the precision of species identification in future studies on ergasilid diversity worldwide.

## Data Availability

Voucher material deposited in the Copepoda collection of the Instituto de Ciencias del Mar y Limnología, Unidad Académica Mazatlán (ICML-EMUCOP), Sinaloa, Mexico. Sequence data have been deposited in GenBank (accession coded PP239046, PP239047, PP239048).
